# Cultural Identity Among Afghan and Iraqi Traumatized Refugees: Towards a Conceptual Framework for Mental Health Care Professionals

**DOI:** 10.1007/s11013-016-9514-7

**Published:** 2017-01-20

**Authors:** Simon P. N. Groen, Annemiek Richters, Cornelis J. Laban, Walter L. J. M. Devillé

**Affiliations:** 1 De Evenaar, North Netherlands Centre of Transcultural Psychiatry, GGZ Drenthe Mental Health Care, PO Box 30007, 9400 RA Assen, The Netherlands; 2 Amsterdam Institute for Social Science Research, University of Amsterdam, Amsterdam, The Netherlands; 3 Faculty of Social and Behavioral Sciences, University of Amsterdam, Amsterdam, The Netherlands; 4 National Knowledge and Advisory Center on Migrants, Refugees and Health (Pharos), Utrecht, The Netherlands; 5 Julius Center for Health Sciences and Primary Care, University Medical Center Utrecht, Utrecht, The Netherlands

**Keywords:** Cultural identity, Refugees, Posttraumatic stress disorder, Acculturation, Grounded theory

## Abstract

Cultural identity in relation with mental health is of growing interest in the field of transcultural psychiatry. However, there is a need to clarify the concept of cultural identity in order to make it useful in clinical practice. The purpose of this study is to unravel the complexity and many layers of cultural identity, and to assess how stress and acculturation relate to (changes in) cultural identity. As part of a larger study about cultural identity, trauma, and mental health, 85 patients from Afghanistan and Iraq in treatment for trauma-related disorders were interviewed with a Brief Cultural Interview. The interviews were analysed through qualitative data analysis using the procedures of grounded theory. The analysis resulted in three domains of cultural identity: personal identity, ethnic identity and social identity. Within each domain relationships with stress and acculturation were identified. The results offer insight into the intensity of changes in cultural identity, caused by pre-and post-migration stressors and the process of acculturation. Based on the research findings recommendations are formulated to enhance the cultural competency of mental health workers.

## Introduction

Cultural identity in relation to mental health is of growing interest in the field of transcultural psychiatry. In 1999, Bhugra et al. stated that clinicians underestimate the relation between cultural identity and mental health, and concluded that there is an urgent need for placing cultural identity back at the core of the individual’s well-being. Similarly, but more recently, attention has been drawn to the need for health professionals to take cultural identity into account when trying to understand social and individual functioning of migrants with mental health problems (Mezzich et al. 2009a). The most obvious way in today’s cultural psychiatry to address that need is the use of the Outline for Cultural Formulation (OCF) in the fourth edition of the Diagnostic and Statistical Manual for Mental Disorders (DSM-IV, American Psychiatric Association 1994), which holds cultural identity as its first component. Cultural identity ‘… serves as an introduction to the rest of the Cultural Formulation’ (Lewis-Fernández and Díaz 2002:278), which includes cultural explanations of the individual’s illness, cultural factors related to the psychosocial environment and levels of functioning, cultural elements in the patient-clinician relationship and an overall cultural assessment. In the OCF, cultural identity is divided into three subheadings: individual’s ethnic or cultural reference group(s); degree of involvement with both the culture of origin and the host culture (for immigrants and ethnic minorities); and language abilities, use, and preference (including multilingualism, Lewis-Fernández 1996:137).

Although cultural identity is frequently employed with respect to culturally diverse patients, a complete or precise understanding of what is understood by cultural identity is hard to find. There have been several elaborations of cultural identity after the one in the OCF, of which two are mentioned here. For some authors cultural identity includes more than the OCF implies: ethnicity, race, country of origin, language, acculturation, gender, age, sexual orientation, religious or spiritual beliefs, and socioeconomic class and education (Lu et al. 1995; Ton and Lim 2006). Basing cultural identity on a list of aspects only is a so-called “trait list approach”, which risks stereotyping and is avoided in ethnography (Kleinman and Benson 2006). Our ethnographic approach to cultural identity focuses on norms and values that constitute an image an individual holds of him or herself, which urges an individual to decide what is right or wrong, what kind of behaviour is appropriate or not; as well as on norms and values that are negotiated within the (ethnic or ethnoreligious) group the individual belongs to; and within local society. In our view, cultural identity includes a broad range of ethnic and social characteristics, which may be unique in each individual’s situation and his or her perception of this situation. These characteristics are often underexposed in mental health care. In the case of refugee patients, cultural identity could be regarded as fluid, multiple, ever-changing, a *perpetuum mobile*, in a certain microcosm, especially changing through potentially traumatic events (PTEs), migration, and, as a consequence, acculturation into a new society (Rohlof, Knipscheer, and Kleber 2009; Ton and Lim 2006; Bhugra 2005).

For immigrants, cultural identity is inclined to change in the process of acculturation in the host society. The impact of acculturation on people’s cultural identity alone may contribute to stress among refugees (Bhugra 2004; Bhugra and Becker 2005). Acculturation refers to the degree of identification with the host culture and/or with the culture of origin. Positive identification with both cultures among immigrants results in lowest risk for mental distress, while negative identification with both cultures leads to the highest risk (Fassaert et al. 2011; Kamperman et al. 2003). It should be noted that refugees distinguish themselves from general immigrants, because most were forced to leave their country of origin and the majority experienced PTEs which cause higher risk for mental health problems (Gerritsen et al. 2006; Laban et al. 2005; Porter and Haslam 2005).

Traumatic stress also has a strong impact on cultural identity, because experiencing one or more PTEs, and also loss, grief, and bereavement that result from PTEs, may have a devastating effect on identity development. The memory of PTEs tends to form a cognitive reference point for the organization of other memories leading to an enhanced integration of these PTEs in a person’s understanding of him- or herself and the world (Berntsen and Rubin 2007:427). This personal understanding may vary between different cultures, for instance between so-called independent and interdependent cultures, as Jobson and O’Kearney (2008) have shown in a study among immigrants suffering from posttraumatic stress disorder (PTSD).

Because traumatic memory is so central for a person’s identity, crucially informed by culture, clarity about the patient’s cultural identity is expected to be necessary for most types of trauma treatment and crucial for mental health care professionals (Bäärnhielm and Scarpinati Rosso 2009). The elaboration of the concept of cultural identity increases the need for further clarification of the concept for clinicians in their clinical encounter with culturally diverse patients. The ability to profit from insight in cultural identity for better understanding of the illness seems to depend on the cultural competency of the clinician. A challenge for cultural competency is overcoming differences in cultural knowledge and various items of cultural identity (Kirmayer 2012; Kleinman and Benson 2006). For trauma survivors, all the personal and social losses, grief, and bereavement that result from PTEs and migration are inextricably connected to identity (Eisenbruch 1991). Reducing the meaning of a patient’s story to psychiatric symptoms may increase the risk of misunderstanding between patient and clinician and systematic misjudgements in diagnosis and treatment (Lewis-Fernández and Díaz 2002).

### Aim of the Study

The aim of this study is to unravel the complexity and many layers of cultural identity in traumatized asylum seekers and refugees in mental health care, to assess how stress and acculturation relate to (changes in) cultural identity and how cultural identity can be organized into domains that could be useful for mental health workers in their diagnosis and treatment of those patients. To contextualize the results of this study that took place in the Netherlands, the reader should know that in the research period, islamophobia was increasing with the consequence that all migrants were under pressure to assimilate quickly, especially newcomers. More recent the DSM-5 Cultural Formulation Interview (CFI, American Psychiatric Association 2013) was introduced. This interview includes three questions in its core version on cultural identity as well as a supplementary module on this topic. This study was conducted prior to the introduction of the CFI. We will refer to this latest development in the discussion section.

## Methods

In a cross-sectional design patients who were referred to a Dutch Centre for Transcultural Psychiatry ‘De Evenaar’ were interviewed using a Brief Cultural Interview (BCI; Groen et al. 2016). The BCI is a standard part of the diagnostic assessment of the centre. It is a semi-structured questionnaire based on the DSM-IV’s OCF domains that are listed above. The BCI is used in a narrative fashion, in which the 27 questions serve as a guideline leaving space for dialogue, in line with methodological recommendations for use of the OCF that include a graceful flow of questions and answers, an open inquiry, and an attitude of empathic concern (Mezzich et al. 2009b). The aim of this methodological approach is to stimulate clinician-patient interaction. Eleven questions concern cultural identity (see “Appendix” section). In line with Kleinman and Benson (2006) we opt for an ethnographic approach of a more encompassing understanding of cultural identity than a trait list approach. An ‘emic’ account of cultural identity was a result of the dialogue between patient and interviewer. All interviews were conducted in the Dutch Centre by the same anthropologist (first author). They were not tape-recorded. The interviewer reconstructed interview reports post hoc. Ethical approval has been obtained at the University Medical Centre of the University of Groningen (2012.404).

### Sample

The BCI’s were conducted among 43 Afghan and 42 Iraqi refugee patients from February 2006 until April 2011. All patients were diagnosed with PTSD, and/or Anxiety and/or Depression Disorder. To prevent too much cultural diversity in the study group, only adult respondents from Iraq and Afghanistan were selected. They represented the largest groups of asylum seekers and refugees in the Netherlands at the time. In the Netherlands, asylum seekers await the decision of granting a residence permit in asylum seekers centres. Refugees are residence permit holders. Patients with a psychotic disorder or substance-related disorders were excluded. Among the respondents, 34 Afghans needed a Dari interpreter, while 30 Iraqis required an Arabic (21), Sorani (7), or Turkish (2) interpreter. Only official interpreters were used (TVcN Interpretation and Translation Centre of the Netherlands). All other interviews were conducted in Dutch. It was checked whether fluency in Dutch was sufficient during the earlier psychiatric assessment. No reticence of female Muslim patients towards the male interviewer was noticed. Apart from age and gender we registered ethnic group, place of birth, religion, length of stay in the Netherlands, refugee status, and family situation (Table 1).

**Table 1:** Socio-demographic characteristics of Afghan and Iraqi patients under treatment between 2006-2011

	Afghans	Iraqis	Total
	*n* = 43	*n* = 42	*n* = 85
Age			
Mean	35 (SD = 12.74)	37 (SD = 8.82)	36 (SD = 15.05)
Range	17–81	17–58	17–81
Gender			
Male (%)	44.2	59.5	50.6
Female (%)	55.8	40.5	49.4
Ethnicity			
Tajik	15		
Pashtun	5		
Hazara	5		
Arabic		23	
Kurdish		13	
Turkmen		3	
Mixed*	3		
Other	5	3	
Religion			
Muslim**		16	16
Shiite	14	10	24
Sunni	10	4	14
Mixed***		2	2
Christian	3	3	6
Not known****	16		16
Family			
Single	5	7	12
Single with children NL	3	1	4
Single with family NL	5	4	9
Partner NL, no children	1	1	2
Partner abroad, no children	1	0	1
Partner and children NL	24	19	43
Partner and children abroad	3	5	8
Partner and children NL/abroad	1	3	4
Divorced with children	0	2	2
Juridical status			
Asylum (%)	66.7	42.9	54.8
Refugee (%)	33.3	57.1	45.2
Stay in NL (years)*****	4.44	5.51	4.96
Male	6.11	5.52	5.77
Female*****	3.00	5.50	3.97

* One of the parents was Tajik, the other Pashtun

** Number of patients who did not want to say whether they were Shiite or Sunni

*** One of the parents was Shiite, the other Sunni Muslim

**** Respondents either did not want to say, or it was not asked

***** The length of stay in the NL of two female patients from Afghanistan and three female patients from Iraq was not known

### Qualitative Analysis

A grounded theory approach was used for the qualitative analysis of the narrated data (Glaser and Strauss 1967). The main thrust of grounded theory is to generate theories regarding social phenomena and to develop a level of understanding that is grounded in a systematic analysis of data (Lingard et al. 2008). From the collected data in the 85 BCI reports a series of open codes assigned to narrative reconstructions were identified based on our ethnographic definition of cultural identity, using Atlas.ti, version 6.2 (ATLAS.ti Scientific Software Development GmbH, Berlin). Open coding means that in the reports first cultural identity items were broadly distinguished. This resulted in a list of codes that were further assigned to narratives in subsequent BCI reports. Codes were added until the point of saturation was reached. The coding process was then refined by further analysis of the narratives and detecting additional cultural identity items. The codes were analysed and then clustered into subgroups, shifting back-and-forth between codes and narratives (Gibbs 2007). Next, subgroups were grouped into domains of cultural identity that form the basis of the conceptual framework. Finally, for each item per domain the relation with stress and acculturation was deducted from the interviews. A topic referring to a stressful event (e.g., forced marriage) was connected with a designated identity item (e.g., among adolescents). The same procedure was followed when a problem was clearly linked to acculturation (e.g., illiteracy hampering integration—women were not allowed schooling).

## Results

The initial coding process of the interviews in Atlas.ti resulted in 70 different codes. Redefining and clustering the codes led to 56 codes (e.g., education of the children in the Netherlands, education in the home country, and education in the Netherlands were clustered into ‘education’). These codes were aggregated in 18 subgroups (e.g., Dutch, mother tongue, other languages, languages among children and literacy were categorized as ‘language’). These subgroups were further classified into three main domains of cultural identity each containing six items: 1) personal identity (age, gender, marital status, education, work, social class/position); 2) ethnic identity (ethnic values general, ethnicity problems, ethnoreligious problems, language, political activity, physical features); and 3) social identity (family, role/position within family, social status, social relations, relationships, social contacts). Each of the domains represents the current personal situation of patients at the time they were interviewed and cultural connotations as they were conceived by the individual, by others in the same ethnic group, and/or by others in local society. For example, ‘age’ is not only biological age, but concerns the cultural meanings that are attached to an individual of a certain age, expectations of a certain age in a specific ethnic group, and local norms and values attached to that age in contact with others. Similarly, in an ethnographic understanding of cultural identity, ‘gender’ is regarded as meanings an individual ascribes to a sense of femininity or masculinity for her or himself, as a female or male member of an ethnic group, and in relation to others beyond the ethnic group.

Tables 2, 3 and 4 present each one of the three domains of cultural identity and their specific relation to stress and acculturation for each item. The cultural aspects in all tables should be regarded as examples, not as generalizations that apply to every respondent in every situation at any given point in time.

**Table 2:** Personal identity: narrated cultural identity items divided into personal items in interviews with Afghan (n = 43) and Iraqi (n = 42) patients

Items	Subdivision	Relation with stress	Relation with acculturation
Age	Childhood	Unaware of flight reason	Living between two cultures
	Adolescence	Forced marriage, kidnap	Differences with Dutch peers
	Adults	Exposure to PTE (war periods)	Feeling disconnected from Dutch society
Gender	Women	Vulnerability of women Forced to stay at home No schooling	Wanting same rights as Dutch women Ambivalence towards more freedom for women Illiteracy hampers communication
	Men	Kidnap from work or failure in male duties Unable to fulfill duties Partner/father role	Differences between men and women in NL Decreased sense of masculinity Increased distance to children and partner
Marital status	Single Married Widowed	Loneliness Mixed marriage Stalking single/widowed women	Failing to connect to Dutch peers Difference between partners in adaptation to NL Feeling unsafe among nationals
Education	Low Middle High	Difficulty establishing position society Disparity with Dutch middle educated Loss of education achievement Threats by Taliban	Difficulty establishing position in NL Feeling distance to Dutch peers Feelings less appreciated in NL Feeling less worth than Dutch high educated
Work	Low class Middle class High class	Difficulty finding new job Fear of losing one’s job Unable to regain similar level of work	Feeling powerless in NL Feeling different than Dutch workers Dissatisfaction with work in NL
Social class/position	Low Middle High	Vulnerability Feeling bereft of social position Loss of social status	Feeling unable to participate in NL Feeling unable to profit from opportunities offered in NL Feeling worthless compared to home society

**Table 3:** Ethnic identity: Narrated cultural identity items divided into ethnicity items in interviews with Afghan (n = 43) and Iraqi (n = 42) patients

Items	Subdivision	Relation with stress	Relation with acculturation
Ethnic values general	Values Upbringing	Stress in relation to other ethnic groups Fear of other ethnic groups	Avoiding contact with other ethnic groups Discrimination by other ethnic groups
Ethnicity problems	Ethnic group Ethnic position Ethnicity parents Ethnicity partner	Tensions with another ethnic group Oppressed as ethnic minority Mixed ethnic/religious groups led to threats Other ethnic/religious group led to distress	Avoiding contact with other ethnic groups Feeling marginalized in NL Not belonging to either parent’s groups Not being accepted by own/partner group
Ethnoreligious problems	Religion home Religion NL	Fear of other religious groups Fear other religions, conversion to Christianity	Avoiding contact with Iraqis/Afghans Avoiding contact with Sunnis/Muslims
Language	Dutch Mother tongue Other languages Children self Literacy	Fear of not being understood Fear to speak in public (Kurdish) Threat because of mixed marriage parents Not being able to understand children Fear of not being able to learn Dutch	Perception of not being understood in NL Unable to speak to other nationals Not feeling accepted by nationals Fear of children loosing their mother tongue Less able to learn Dutch compared to others
Political activity	Activity home Military home Political situation	On black list because of ethnic power changes Ethnic and religious discrimination in army Trauma due to political change home country	Wanting to stay quiet/under cover in NL
Physical features		Ethnicity is easily recognized Shame, withdrawal, avoidance	Not being accepted by other ethnic groups in NL Not wanting (much) social contact in NL

**Table 4:** Social identity: Narrated cultural identity items divided into social items in interviews with Afghan (n = 43) and Iraqi (n = 42) patients

Items	Subdivision	Relation with stress	Relation with acculturation
Family	Home now Home then Family NL	Worries about family in the home country Murdered/killed/missing family members Worries about family in NL	Thinking more about home than about NL Feeling distant to Dutch with complete families Losing norms, values and mother tongue
Role/position within the family	Father role Mother role Position family	Feeling unable to fulfill father tasks Feeling to fail as a mother Feeling powerless, meaningless	Avoiding social contact in NL Avoiding social contact in NL Feeling to fail in NL
Social status	Profession parents Profession partner Social position NL	Social drop, negative change of social status Fear for risks of high status partner Marginalized, feeling guilty, single woman	Loss of social status in society Feeling lost in NL Trapped between culture NL and home society
Social relations	Children home Children NL Parents home Parents NL Other family	Children missing, children in home country Stress about health situation child Worries about parents in home country Stress with parents concerning upbringing Fear of family in law, lack of support	Failing as parent hampers integration Language problems with children Feeling lack of support in NL Differences in acculturation with parents Differences in acculturation with other family
Relationships	Partner home Partner NL	Missing partner, stress about safety partner Relationship problems, overloaded with tasks	Loneliness, women afraid of men in NL Loneliness, acculturation differences partner
Social contacts	Children Family home Family in NL Fellows In NL With Dutch	Stress about friends of children in NL Grief over parents, lack of establishing contact Few contact because of stress within the family Ethnic problems, afraid of curiosity Acculturation stress Not sufficient concentration to learn Dutch	Dependent on children for communication Homesickness Family more important than integration Avoiding contact with peer group Experiencing social distance to the Dutch Few opportunities to communicate in Dutch

### Personal Identity and Relation with Stress and Acculturation

In the BCI reports, cultural items of personal identity depict the personal situation in the home country, often related to stress, and imply change through the acculturation process in the host society. Combinations of these personal identity traits make an individual unique compared to others.

Age is divided into life stages (child, adolescent, adult) that relate to certain PTEs in the country of origin. When PTEs have been experienced within the family during a respondent’s childhood resulting in flight, the main relation to stress in the reports is the unawareness of the flight reason, because trauma is silenced. Young respondents often experience an unclear cultural identity, because they feel not to belong to the culture of origin, neither to the host culture. The main relation to stress for adolescents is kidnapping for boys and forced marriage for girls:

After the death of my father I was sent to a mosque when I was between 10 and 12 years of age. After that I kept myself busy with traditional sewing and embroidery. At the age of 16 I married my husband, whom my grandfather and the oldest village inhabitants had identified as a suitable spouse for me. I could not oppose to that marriage, because as a girl I was not entitled to do so. (Afghan woman, 31 years old, interview in Dari with interpreter)

Many respondents experience difficulties identifying with Dutch peers, who grew up with different norms and values regarding freedom of choice (e.g., marriage, study) and negligible risk of being kidnapped or other violence (e.g., “they do not know the dangers in my country, that is why I am different”). There may be several cultural differences in life stages (e.g., age to marry, to have children). For adults, exposure to PTEs in general has caused stress. Many respondents fail to connect to Dutch society, because of mental health problems or conflicting norms and values.

Gender distinguishes male and female respondents in relation to stress and to acculturation problems. The example of a 23-years old Sunni Pashtun woman who at the age of six fled from Kabul to Jalalabad in the far east of Afghanistan and ran away from forced marriage points out that a forced flight in childhood changed her perspective on norms and values within her country concerning the role of women:

My earliest memory is that of being a witness to murders on the street, hiding in a safe place and sometimes having nothing to eat for days. The move to Jalalabad was a great cultural shock, because women were not allowed free choices by the Taliban. One of the consequences was that my mother had to stop working. I felt that women were treated as animals, unlike in Kabul where women had more opportunities. I asked my parents many times why things changed, without getting any answer. At the age of nine I had to stop school. I tried to pursue my education at home while being responsible for the entire household as I was the eldest daughter and my mother suffered from severe diabetes. (Interview in English)

The stress of failing as a Muslim daughter and a role model for her younger sisters, is accompanied by experiencing difficulties identifying with Dutch peers for whom equal rights for women is the norm. Many girls in Afghanistan were not allowed schooling, which complicates their acculturation in Dutch society because of illiteracy and a cultural gap between less and more freedom for women. Girls in Iraq are more often educated, although there may be differences between Arab and Kurdish girls and they are mostly not as much educated as their Dutch peers. Afghan men’s main relation to stress in the home society is being kidnapped because of their work for the government, or because they could not “control their wives”. In Iraq, many male respondents suffer from stress because of changes after the fall of the Saddam Hussein regime in 2003. They feel unable to fulfil their duties as a partner and/or a father, which affects their cultural sense of masculinity, and leads to emotional distance to their nuclear family. Failing as a partner and/or a father also results in a lack of self-confidence to integrate into Dutch society.

Marital status carries cultural connotations attached to roles that are rooted in all kinds of local attitudes to, for example, being single or widowed, and conflicts, for instance related to mixed ethnic or religious marriages. Many single or widowed Afghan women experience stress being stalked by Afghan men. Consequently, they feel unsafe and do not want to go out of the home, which hampers participation in Dutch society. Partners of ethnically or religiously mixed marriage experience differences in level of acculturation: mostly the wife integrates more easily.

The level of education and the kind of work include deeply rooted cultural expectations associated with pride, shame, feeling (in)secure, and being a meaningful person in society:

In the Netherlands I first went to school in the asylum seekers centre to learn the language. I had no contact with other Iraqis. After six months we moved to another city, where I first entered an international class and then a class to prepare for entering a Dutch school. Contact with the other children was difficult. I had another mentality and my way of thinking was different from my classmates. They had things, while I did not. (Iraqi man, 27 years old, interview in Dutch)

Those respondents with little or no educational attainment may find it stressful to establish a new position or job in Dutch society and experience a cultural gap with the Dutch. In the respondents’ sample, Afghans had often less educational attainment compared to Iraqis. Middle class workers are afraid to lose their job, and high class workers are unable to regain the same or a comparable level of work in the Netherlands. This results in acculturation problems of feeling powerless, feeling different from Dutch colleagues and dissatisfaction in Dutch society.

### Ethnic Identity and Relation with Stress and Acculturation

Cultural items of ethnic identity were specifically related to pre-migration stress in the BCI reports. Ethnicity refers to problems respondents encountered because of their belonging to a specific ethnic group, especially to an ethnic minority, in their home country. Religion is classified under ethnicity because, mostly, it distinguishes one ethnic group from the other, although religion is not equivalent to an ethnic group.

General ethnic values concern values that distinguish one ethnic group from the other, in some cases differentiated in the upbringing of the self and/or the children. Problems that have arisen may have an extensive history of ethnic conflict, as the case of a 41-year old Kurdish woman illustrates:

My first memory of my Kurdish identity was the planes of Saddam that I saw flying over when I was ten years old. Bombings followed, that made my family run into the mountains. I have also been a witness to the beheading of my neighbour’s son. The neighbours cried. When I was 17 or 18 years old I joined in demonstrations against the Arab ruler and donated blood to the victims. A friend of mine had to go to prison. I worked for the Kurds a lot and I worked as a teacher in a boy school. Because of those experiences I hate Saddam and Arab people. At school all children had to cheer “long live Saddam”, but nobody wanted to. You always had to be careful what to say. There was no freedom of speech. One day a teacher was taken away by “a car from Saddam”. I never saw that teacher again. It came out that the cleaning lady from school worked for Saddam. (Interview in Dutch)

These general ethnic values implicated stress in the home country because of political problems, that affect the ethnic identity, but not for all respondents. In some cases, ethnic problems continue in the host country. A considerable number of respondents avoid social contact with nationals who belong to another ethnic group and some report still feeling discriminated in the host country. For a few, this avoidance means having no social contacts with nationals at all, because they are the only person from a specific ethnic group in a village in the host country.

Ethnic problems between groups are often the reason for flight, in some cases because of mixed ethnicity within the family. These problems lead to stress in the shape of (death) threats and suppression. Many Hazara respondents from rural areas have been confronted with regular conflicts with local Taliban, who claim that Hazara are not true Afghans:

There were many Pashtun around, who sometimes abducted [Hazara] boys, who were then raped. They had to give money and food to Pashtun. Pashtun, who were all Taliban, vituperated and ridiculed Hazara. Villagers could not pass through Pashtun territory. Once, twelve Hazara were killed when they accidently encountered Pashtun. (Afghan man, 21 years old, interview in Dutch)

‘Why am I born as a Hazara?’ one other respondent asked. Avoidance of social contact with other ethnic nationals in the host country is being expressed as feelings of marginalization and non-belonging.

Language is a category in the narratives that contains mother tongue, other languages, Dutch, language between parents and children, and among children themselves, and literacy. Stress in relation to language comes to light at the level of fear for lack of understanding in the interviews:

It bothers me that I do not speak Dutch well. I prefer not to have contact with the Dutch, because I am afraid that they will think that my Dutch is bad. (Iraqi woman, 40 years old, interview in Dutch)

Language problems lead to all kinds of communication problems in the host country, also within the nuclear family. Several respondents complained about not being able to understand their children.

Military and political activity are included in ethnic identity, because power in both countries changed to another ethnic group, which caused severe problems for some ethnic groups. Political change in the home country arouses stress for specific ethnic groups, especially for ethnic minorities who suffer from ethnic discrimination. In their acculturation process, if ethnicity was a problem in the home country, most are hesitant in, or refrain from social contacts.

Ethnoreligious problems play a significant role in the cause of PTEs for many respondents, which can be at odds with a presumed overarching cultural identity and lead to frustration, fear, and sometimes conversion in the host country. In Afghanistan, mostly Shiites suffer from ethnoreligious violence, especially Hazara who can be recognized by their physical features. In Iraq, violence could be directed towards ethnoreligious minorities such as Christian minorities, and Sunnis, in some cases mostly concerning high functioning government officials. Remarkably, some Iraqis in the study sample claimed they were “just Muslim”, and tended to distance themselves from the political divide between Shiites and Sunnis, while Afghan respondents did not. Fear of other religious groups continues in the host country, but to a lesser extent, except for those who converted to Christianity. In many cases, this fear leads to avoidance of social contacts with other ethnoreligious groups.

### Social Identity and Relation with Stress and Acculturation

Cultural items of social identity express the transformation of respondents as social beings in relation to relevant others. In the reports, cultural items of social identity were numerous, with emphasis on social loss in the host society leading to stress and acculturation problems.

Family and social relations, at the time of residence in the home country, and in the host society, are extremely important in the BCI reports. Many respondents describe having problems living without their family, not being able to contact family members, or not even knowing where they are, as missing parts of themselves. Many single young males from Afghanistan declared that living without their family is “having no life at all”. The relation to stress consists of worries about the family in the home country, who might have been murdered, killed in action, or missing family members while residing in the home country and worries about change of overall well-being or mental health of various family members in the host country. This stress leads to ‘mental absence’ in the host society, feelings of being different because most Dutch families are complete. It also results in worries about the prosperous acculturation of children, who are losing their mother tongue. These children adapt to Dutch norms and values more easily, which may lead to a cultural distance to their parents.

Within the family, respondents experience a depreciation of their social roles (e.g., being a father). In particular, being the eldest son is conceived as troublesome because of responsibilities towards the family, the risk of failing to meet social obligations, and not being a good role model for other family members:

I tried to take care of my younger brother and sister as good as possible. I tried to let my little brother go to school, because I could not. I was and I am responsible for them. (Afghan man, 22 years old, interview in Pashto with interpreter)

Being a son or a daughter is sometimes crucial for one’s cultural identity, because the father preferred a son over a daughter, or a mother is being laughed at for only having daughters. Stress is perceived as feeling unable to fulfil tasks, powerlessness, to fail, or meaningless.

Social status depends on the profession of the parents and/or the partner in the home country, and the social position in the host country. Many women experienced stress that is connected to a high social status of their partner, while in a minority of cases a high social status of the wife was subject to stress for the husband. Stress of feeling at the margin, feeling guilty, or having to live as a single woman because of the murder of one’s husband due to his social status is experienced among both Afghans and Iraqis. Concerning their social position in the host country, many respondents feel deprived of their social status, lost in the host society, and/or trapped between two cultures.

Intimate relationships have altered in many cases. For some respondents, stress concerns missing their partner who is killed or left in the home country, or, in some cases, stress about the safety of their partner. For those respondents with a partner in the host society, stress concerns relationship problems, and/or being overloaded with household tasks, because their partner is (mentally) ill. In most cases, these problems result in loneliness. When female respondents experienced PTEs such as sexual trauma, fear of men continues in the host society. Male respondents more often fear to fail with regard to participation in the host society in comparison with their wives.

Lower level of social contacts (e.g., family, peers, Dutch) leads to stress both within and outside the family. Parents often feel dependent on their children for communication in the host society. Worries about the family in the home country often lead to homesickness:

Following the news makes me sad, because then I think of my family. I cannot go to sleep. Already a car bomb has exploded in front of my house there. All windows were broken… Physically I am in the Netherlands, but mentally I am in Iraq. I feel very lonely, because there is nobody who sympathizes with me. I wonder how long I will be separated from my family. This has made me desperate and confused. To be there means death, to be here means being without my family. That is why I hate life. (Iraqi man, 41 years old, interview in Arabic with interpreter)

Respondents experience stress in social contacts with different ethnic groups, due to acculturation stress, and concentration problems that hamper learning Dutch. Many respondents experience social distance to the Dutch, because of differences in norms and values such as pride, respect, and other relevant issues.

## Discussion

Thorough analysis of cultural identity in 85 interviews with Afghan and Iraqi refugee patients resulted in a subdivision of personal, ethnic, and social identity that is potentially relevant in intercultural encounters. Moreover, the extent to which each identity relates to stress and to acculturation problems points at the relevance of understanding various aspects of cultural identity in relation to mental health problems. The results of this study therefore support the inclusion of cultural identity in the Cultural Formulation Interview in DSM-5 (American Psychiatric Association 2013) and a supplementary module (Lewis-Fernández et al. 2016). The supplementary module on cultural identity, which is not based on empirical results, also includes national and racial background, spirituality, religion and moral traditions, and gender and sexual orientation identity, which were not particularly found in this study, except for ethnoreligious problems. More research in different settings among different populations is needed, but reasons for underutilization of the DSM-IV OCF, due to limited dissemination efforts towards practicing clinicians and time required for its implementation (Lewis-Fernández 2009), indicates that a simplified framework could enhance utilization. In case the particular situation of a specific patient requires more elaboration, for instance in the case of problems related to race or sexual orientation, mental health professionals could opt for the supplementary module.

Personal identity offers the clinician information about the personal characteristics and circumstances of the individual patient that are subject to change under the influence of stress and acculturation. Results regarding ethnic identity show how crucial ethnic belonging is to stress in the home country. Around social identity, stress is felt around the family, both in the home and in the host country, inability concerning social expectations within the family that have altered, and the diminished level of social functioning, for example by stress in social contacts. Social identity is of particular interest for this group, because of migration from an interdependent society to an independent society which, in many cases, leads to feelings of alienation. The uniqueness of the findings complicates comparison to other research, because used concepts might contain other elements than found in this study.

### Interaction of Personal Identity with Stress and Acculturation

Experienced PTEs result in an affected integrity of cultural personal characteristics of the refugee patients in this study. There may be typical life threatening risks specific for a culture that affect the kind of person someone believes to be, for example regarding safety for children and women, but also for men who are not able to fulfil duties according to local norms in the society of origin. Change in personal identity is certainly due to the transition from an interdependent to an independent society, which is often quintessential for recovery from stress (Jobson and O’Kearney 2008). However, our results indicate how loss of personal achievements affects the individual. Stress and acculturation impact personal identity in three main ways. Firstly, chronic stress reinforces difficulties for successful integration of the individual. Personal identity was relatively clear in the society of origin, but when loss, grief, or bereavement have affected that clarity, confrontation with norms in the host society causes an even more unclear personal identity. Secondly, difficulties in the acculturation process, on their turn, seem to reinforce stress at the personal identity level, because of cultural differences refugee patients encounter in contact with the Dutch. Thirdly, loss of education achievements, work and social status deprive a person of self-esteem and being a meaningful person in the host society. On a personal level, respondents in this study appear to acknowledge that “mental disorder inevitably challenges traditional ideas about personal identity since, as the notion of disorder suggests, it can profoundly alter and transform its sufferer, disrupting the smooth continuity uniting earlier and later parts of subjectivity and, viewed from the outside, of persons and lives” (Radden 2004:133). The results of our in-depth study show *how* stress and acculturation may alter and transform traditional ideas about personal identity through the items in Table 2. The ubiquity of stress caused by PTEs in thinking about the self as a meaningful person is clarified through a variety of personal roles in various new situations.

### Interaction of Ethnic Identity with Stress and Acculturation

In the respondents’ stories, ethnicity plays such an important role in suffering from PTEs, stress and acculturation problems, that ethnic identity should be distinguished from social identity. Theoretically, ethnicity might be considered a special kind of social identity (Schwartz et al. 2006), but our study shows that special attention to ethnicity would enhance understanding of suffering, coping, and acculturation of, in this case, traumatized refugee patients. Ethnic identity has been considered “… a crucial facet of an individual’s overall cultural identity” (Ton and Lim 2006:10), but the distinguished items of ethnic identity in this study indicate *how*, for instance, being Kurdish in Iraq or a Shiite Tajik or Hazara in Afghanistan may be crucial for vulnerability to PTEs. Our study results underline earlier findings of vulnerability to exposure to PTEs and a higher risk of developing mental health problems in ethnic minorities (McKenzie 2008; Khaylis et al. 2007; Vega and Rumbaut 1991) and show how their ethnic identity is affected, mainly through discrimination. Poor adaptation to the host society among affected ethnic minority groups is an additional risk factor for mental health problems, because a low, weak, or diffuse ethnic identity is related to low self-esteem and psychological well-being (Phinney 1991; Phinney et al. 2001). Our study indicates that the initial conditions for participation in the host society, communication abilities and feelings of being understood, are very poor. Therefore, the results of this study underscore the need to study ethnic identity and acculturation, for ethnic minorities in the host country and/or ethnic minorities among immigrants from the same country of origin (Persky and Birman 2005; Tsai et al. 2002).

### Interaction of Social Identity with Stress and Acculturation

Since social support from relations within and outside the family, social position, and social status are crucial for refugee patients from interdependent societies, which most (post)conflict areas are, social identity ought to be distinguished as a domain of cultural identity. The variety and frequency of items in this domain point out how important social identity is to the respondents and how changes as a consequence of stress and acculturation affect their lives as social beings. Social identity may be crucial for self-esteem, and, it is postulated, maybe even more than personal identity (Taylor and Usborne 2010). The consequences of loss, grief, and bereavement for social functioning in the host society are numerous. The findings in this study underline that the transition to a new society has effected in a dramatic fall of the social functioning of respondents who socialized in interdependent societies, although individuals may be idiocentric or allocentric (Bhugra 2005). Their social embeddedness in the past and stress caused by social loss, grief and bereavement seem to deflate their social self in the present, causing a socio-cultural void. Social bereavement adds to lack of trust in others and avoiding social contacts that are symptoms of PTSD alone, and hamper participation in society. Acculturation problems in social interaction intensify a downturn in social and individual functioning, causing loneliness, homesickness and feelings of being lost in society.

### Implications for Cultural Competency

Elaborating on the suggestion that the concept of cultural identity in DSM-IV needs further development, the framework offers three domains: start from the perspective of the individual (personal identity), amplify it to the potentially most direct meaningful social environment (ethnic identity) and contextualise within the wider social environment (social identity). For mental health professionals this elaboration may help to increase cultural competency. Noteworthy, the health care provider ought to be open-minded, willing to learn about cultural differences, and treat each patient as an individual (Jenks 2011). Open-mindedness towards cultural identity might enhance understanding of the change refugee patients have undergone and lead to what is really at stake for these patients (Kleinman and Benson 2006). Most respondents in this study feel they are bereft of their ‘old’ cultural identity while a ‘new’ cultural identity has not been established yet, partly due to mental health problems and partly due to acculturation problems. Eisenbruch’s definition of cultural bereavement as “… the experience of the uprooted person… resulting from loss of social structures, cultural values, and self-identity” (1991:674) addresses representations of the self and loss in our study results.

For clinicians, we reach the following recommendations to explore cultural identity of culturally diverse patients in their diagnosis and treatment:

- discuss the meaning of personal identity items, such as age, gender, education and work, in the light of their socio-historical context in order to understand the changes PTEs have made to these personal characteristics of the individual;
- discuss the patient’s experiences with one’s ethnic group, with members of another ethnic group, what these experiences mean to him or her, in the past and in the present, and how these are related to causes of mental health problems;
- discuss changes in the social environment, in the family or in socio-economic perspective (decline of social status) and how these changes are related to coping with mental health problems;
- apart from the stress factors from the past, discuss social changes after migration that are often more, or more intense, or more complex than expected at first, and how these changes intensify or influence coping with mental health problems.

Information concerning cultural identity offers clinicians opportunities to a holistic perspective in transcultural psychiatry, from disease to patient to person, on mental health problems in person-centered care (Mezzich et al. 2010). However, they should realize that the provided framework is a simplification of the complexity of cultural identity in daily practice. We recommend mental health care professionals to take the broad range of aspects in the framework as a starting point, but should not limit themselves to them. Above all, they should be aware of the risk of stereotyping (Kleinman and Benson 2006). They should preferably use their patients as their primary source in meaningful dialogue. The focus may even lead to a reshuffling of identity components, but the framework of personal, ethnic, and social identity will presumably not alter.

### Limitations

The results of this study have to be interpreted with some caution. First, the selection of patients from the two largest refugee populations in the Netherlands at the time limits generalization for the entire refugee population. We felt that this multi-ethnic sample is helpful, because from a clinical perspective, clinicians would have more opportunities to practice cultural competency in treatment of patients from various cultural backgrounds then in the case we selected only one country of origin, or one ethnic or religious group. Second, the data are gathered in one transcultural mental health institute in the Netherlands. Comparison with other mental health institutes might have enhanced the findings. Third, we did not distinguish strongly between refugees and asylum seekers in our research because our focus was on cultural identity in all variations, not depending on juridical status. Uncertainty about stay may have had consequences for the acculturation style and language abilities depending on the length of stay in the Netherlands. We will elaborate on differences between asylum seekers and refugees in further research. Fourth, the qualitative analyses have been carried out by the researcher who also conducted the interviews, which may have led to a bias. This was for practical reasons and there has been no other interest than to explore the relevance of cultural identity for clinicians. We acknowledge that theoretical pre-occupations of the researchers which were referred to in the introduction may have driven the results. Finally, the questions concerning cultural identity were leading questions to enhance patient-interviewer interaction, but may have had a steering influence on the outcomes.

## Conclusion

In this article we suggest, based on qualitative research of interview reports collected among traumatized refugees from Afghanistan and Iraq, a framework for cultural identity that can be potentially useful for clinicians in transcultural psychiatry settings. While in dialogue with patients, the complexity of cultural identity may be more appropriately addressed when the clinician takes the division into personal, ethnic, and social identity into account. When clinicians succeed in clarifying changes in these domains as a consequence of stress and acculturation problems, they will have better treatment perspectives, because they could better connect to the culturally diverse patients’ needs, to what is really at stake for these patients.

## Appendix: Questions Concerning the Cultural Identity of the Individual in the Brief Cultural Interview (BCI)

### Language

1. Which language(s) did you speak when you were growing up? Did you also speak another language?
2. Which language(s) do you speak at home now?
  [If applicable: With your wife? With your children? With your friends?]
3. How well do you think you speak Dutch?
  [If this is unclear: How would you grade yourself on a scale of 1 to 10?]
4. Can you explain in Dutch what you mean?

### Ethnicity and Culture

5. Do you belong to a group in your country that is different from other (ethnic) groups?
  Are your parents from the same group?
6. What makes this group different from other groups? Which customs, opinions, position of the group compare to other groups in society?
7. How important is belonging to this group to you?
8. Are you still in contact with people from this group or your culture?
  If so: how important to you is this?
  If not: would you like to?
9. What do you consider to be the important element of your culture?
  [For example: eating customs, respect, family, holidays, honour]
10. How do you think your culture differs from the Dutch customs and opinions?
  Is that important to you?
11. Do you think you fit in well in the Netherlands? Do you talk to Dutch people? Do you have any Dutch friends or acquaintances?
